# Capsaicin Inhibits Multiple Bladder Cancer Cell Phenotypes by Inhibiting Tumor-Associated NADH Oxidase (tNOX) and Sirtuin1 (SIRT1)

**DOI:** 10.3390/molecules21070849

**Published:** 2016-06-28

**Authors:** Ming-Hung Lin, Yi-Hui Lee, Hsiao-Ling Cheng, Huei-Yu Chen, Fong-Han Jhuang, Pin Ju Chueh

**Affiliations:** 1 Institute of Biomedical Sciences, National Chung Hsing University, Taichung 40227, Taiwan; w101052018@gmail.com (M.-H.L.); cornsugar@gmail.com (Y.-H.L.); hikki4028@yahoo.com.tw (H.-L.C.); trista09081123@gmail.com (H.-Y.C.); s7213987@gmail.com (F.-H.J.); 2 Tainan Municipal An-Nan Hospital, China Medical University, Tainan 70965, Taiwan; 3 Graduate Institute of Basic Medicine, China Medical University, Taichung 40402, Taiwan; 4 Department of Medical Research, China Medical University Hospital, Taichung 40402, Taiwan; 5 Department of Biotechnology, Asia University, Taichung 41354, Taiwan

**Keywords:** apoptosis, cancer, capsaicin, silent mating type information regulation 1 (sirtuin1, SIRT1), tumor-associated NADH oxidase (tNOX, ENOX2)

## Abstract

Bladder cancer is one of the most frequent cancers among males, and its poor survival rate reflects problems with aggressiveness and chemo-resistance. Recent interest has focused on the use of chemopreventatives (nontoxic natural agents that may suppress cancer progression) to induce targeted apoptosis for cancer therapy. Capsaicin, which has anti-cancer properties, is one such agent. It is known to preferentially inhibit a tumor-associated NADH oxidase (tNOX) that is preferentially expressed in cancer/transformed cells. Here, we set out to elucidate the correlation between tNOX expression and the inhibitory effects of capsaicin in human bladder cancer cells. We showed that capsaicin downregulates tNOX expression and decreases bladder cancer cell growth by enhancing apoptosis. Moreover, capsaicin was found to reduce the expression levels of several proteins involved in cell cycle progression, in association with increases in the cell doubling time and enhanced cell cycle arrest. Capsaicin was also shown to inhibit the activation of ERK, thereby reducing the phosphorylation of paxillin and FAK, which leads to decreased cell migration. Finally, our results indicate that RNA interference-mediated tNOX depletion enhances spontaneous apoptosis, prolongs cell cycle progression, and reduces cell migration and the epithelial-mesenchymal transition. We also observed a downregulation of sirtuin 1 (SIRT1) in these tNOX-knockdown cells, a deacetylase that is important in multiple cellular functions. Taken together, our results indicate that capsaicin inhibits the growth of bladder cancer cells by inhibiting tNOX and SIRT1 and thereby reducing proliferation, attenuating migration, and prolonging cell cycle progression.

## 1. Introduction

According to the cancer incidence and mortality estimates for 2012, bladder cancer was the sixth most frequent type of cancer among males and the ninth leading cause of death worldwide [1]. Although most patients are initially diagnosed with non-muscle-invasive bladder cancer, many such tumors recur after therapy and eventually exhibit muscle invasion [2]. Cancer is multistage process with a complex etiology. However, the concept of using nontoxic natural compounds to reduce carcinogenesis (called chemoprevention) has emerged as a strategy. Researchers have also recently focused on using such agents against cancer cells to induce apoptosis, which represents a programmed self-killing mechanism that involves extrinsic and intrinsic pathways [3,4,5,6]. In bladder cancer cells, apoptosis is reportedly induced by the ethanol extract of pomegranate fruits [7], the green tea catechin, EGCg [8], grape seed extract [9], and resveratrol [10].

Here, we focused on an active component of chili pepper, capsaicin (8-methyl-*N*-vanillyl-6-nonenamide), which has shown in vitro inhibitory effects on many cancer cell lines [11,12,13,14,15]. The various signaling pathways known to be involved in capsaicin-mediated cellular responses include oxidative stress, which is known to trigger apoptosis [16,17,18,19]. Indeed, capsaicin was shown to induce apoptosis in pancreatic cancer cells in association with reactive oxygen species (ROS) generation and mitochondrial damages [19,20]. However, other pathways are also relevant to capsaicin-mediated apoptosis. Nitric oxide (NO) elevation was found to be induced by capsaicin in association with Mdm2 down-regulation and p53 activation, thereby increases the apoptotic Bax expression and mitochondrial-dependent apoptosis [21]. Capsaicin also exerts its inhibitory effect on the activation of STAT3 in multiple myeloma cells, in turn alters protein expression of Bcl-2, Bcl-xL, and survivin, resulting in apoptosis [22].

We previously demonstrated that capsaicin preferentially targets a tumor-associated NADH oxidase (tNOX) in cancer/transformed cells, thereby enhancing ROS generation and apoptosis [11,12,23,24]. tNOX belongs to a family of growth-related plasma membrane hydroquinone oxidases that are responsible for converting reduced NADH to the oxidized NAD^+^ form [25]. The results from gain- and loss-of-function experiments consistently show a close correlation between tNOX expression and aggressive cancer phenotypes [26,27]. Given that tNOX is associated with cancer phenotypes and is preferentially inhibited by capsaicin, the molecular mechanisms underlying its anticancer properties are of great interest. Capsaicin-mediated ROS overproduction parallels the inhibition of tNOX, suggesting that the latter contributes to the cytotoxicity of capsaicin and might even be an upstream regulator of the intracellular redox homeostasis [12]. In addition, we recently reported that capsaicin diminishes the intracellular NAD^+^/NADH ratio through inhibition of tNOX and subsequent reduction of the NAD^+^-dependent deacetylase, sirtuin 1 deacetylase (SIRT1), which increases p53 acetylation and apoptosis [28]. However, whether tNOX/SIRT1 axis affects cellular functions other than apoptosis has not been examined before.

Here, we present evidence demonstrating that capsaicin-induced tNOX suppression reduces multiple cancer phenotypes in human cancer cells. We further confirm that, for the first time, the downregulation of tNOX concurrently with decreased SIRT1 contribute to the reduced cancer phenotypes, including enhanced apoptosis, increased cell doubling time, and decreased cell migration.

## 2. Results

### 2.1. Capsaicin Downregulates tNOX and Inhibits TSGH8301 Cell Growth by Induction of Mitochondria-Dependent Apoptosis

**Figure 1:**
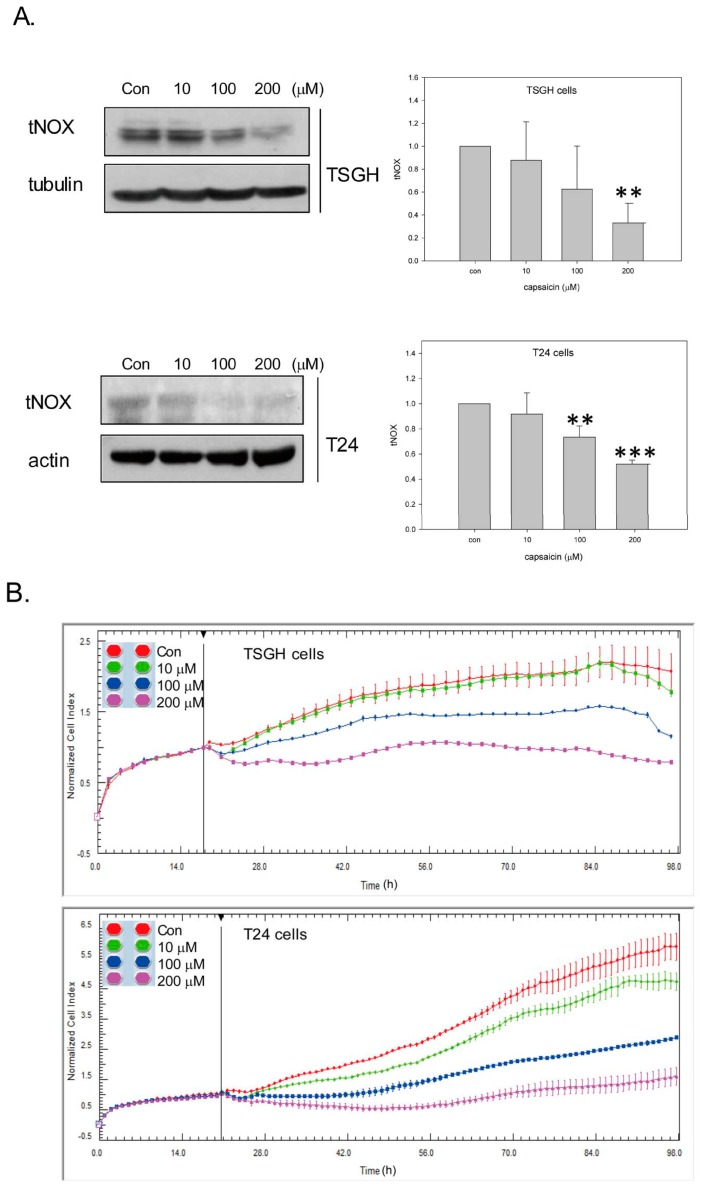
Capsaicin downregulates tNOX expression and inhibits cell growth in TSGH8301 (herein called TSGH) and T24 cells. (**A**) Cells were treated with capsaicin or ethanol for 18 h. Aliquots of cell lysates were separated by SDS-PAGE and analyzed by western blotting; β-actin (or tubulin) was used as an internal control. Representative images from four experiments are shown. The intensity of a protein band was quantified by Gel-Pro Analyzer software 3.1. The resultant values of protein expression were normalized to those of actin. Values (mean ± SE) are from three independent experiments (** *p* < 0.01, *** *p* < 0.001); (**B**) Cell growth with or without capsaicin was dynamically monitored using impedance technology. Normalized cell index values measured over 96 h are shown.

**Figure 2:**
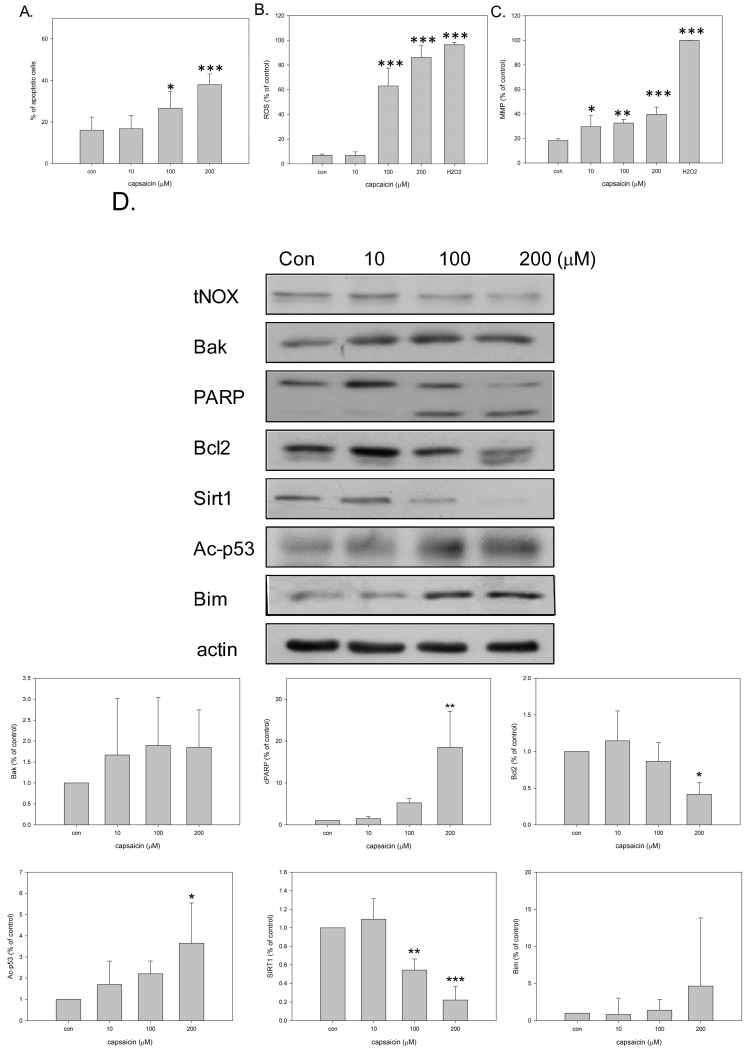
Capsaicin induces mitochondria-dependent apoptosis in TSGH cells. (**A**) Cells were treated with ethanol or capsaicin for 18 h. The distribution of viable (FITC/PI double-negative), early apoptotic (FITC-positive), late apoptotic (FITC/PI double-positive) and necrotic (PI-positive/FITC-negative) cells was analyzed using a Beckman Coulter FC500 flow cytometer. Both early and late apoptotic cells are included in our definition for apoptosis. Values (mean ± SE) are from three independent experiments (* *p* < 0.05, *** *p* < 0.001, for cells treated with capsaicin vs. controls by one-way ANOVA with LSD); (**B**) Cells were treated with ethanol or capsaicin for 6 h. ROS generation was assessed by flow cytometric analysis of H_2_DCFDA in TSGH cells and is expressed as a percentage of cells. Values (mean ± SE) are from four independent experiments (*** *p* < 0.001, for cells treated with capsaicin vs. controls by one-way ANOVA with LSD); (**C**) Cells were treated with capsaicin or ethanol for 3 h. Mitochondrial function was assessed by flow cytometric analysis of JC-10 in TSGH cells and is expressed as a percentage of cells. Values (mean ± SE) are from four independent experiments (* *p* < 0.05, ** *p* < 0.01, *** *p* <0.001, for cells treated with capsaicin vs. controls by one-way ANOVA with LSD); (**D**) Aliquots of cell lysates were separated by SDS-PAGE and analyzed by Western blotting; β-actin was used as an internal control. Representative images are shown. The intensity of a protein band was quantified by Gel-Pro Analyzer software 3.1The resultant values of protein expression were normalized to those of actin. Values (mean ± SE) are from three independent experiments (* *p* < 0.05, ** *p* < 0.01, *** *p* < 0.001).

Capsaicin is found to inhibit tNOX expression in various cancer cell lines, but not in bladder cancer cells. In this study, we found that capsaicin treatment at 100 and 200 μM effectively reduced tNOX expression in bladder cancer TSGH8301 (herein called TSGH) and T24 cells (Figure 1A). As previous studies suggested that tNOX downregulation is associated with reduced cell growth [23,24,27], we monitored the growth of capsaicin-exposed cells by cell impedance measurements. We found that cell growth (presented as normalized cell index values) of TSGH cells was inhibited by 100 and 200 μM, and that of T24 was somewhat reduced by 10 μM of capsaicin (Figure 1B). This cell growth inhibition was accompanied by enhanced apoptosis in TSGH cells treated with 100 and 200 μM capsaicin (Figure 2A). To begin dissecting the underlying mechanisms of this apoptosis, we examined oxidative stress by H_2_DCFDA staining and found that capsaicin dose-dependently increased ROS generation (Figure 2B). JC-10 staining showed that this triggered changes in mitochondrial membrane potential (Figure 2C).

Moreover, protein analysis demonstrated that capsaicin downregulated the pro-survival protein, Bcl2, while upregulating the pro-apoptotic protein, Bak, and enhancing the caspase3-medaited cleavage of PARP (Figure 2D). In this study, we also confirmed the effect of capsaicin on SIRT1 deacetylase in TSGH cells, and found that 100 and 200 μM capsaicin decreased SIRT1 expression and concurrently increased p53 acetylation (Figure 2D).

Previous studies suggest that SIRT1 negatively regulates the expression of the tumor suppressor, FOXO3 [29], and FOXO3 is a key transcription factor for upregulation of the apoptotic protein Bim [30]. Consistent with these, we observed that capsaicin treatments (i.e., SIRT1 downregulation) enhanced the expression level of Bim (Figure 2D).

### 2.2. Capsaicin Downregulates tNOX and Induces Cell Cycle Arrest at G1 Phase

**Figure 3:**
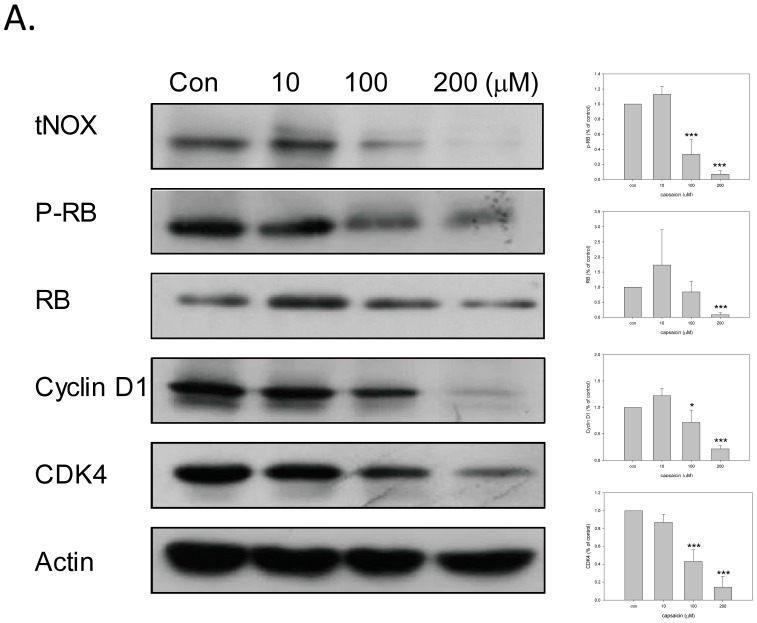

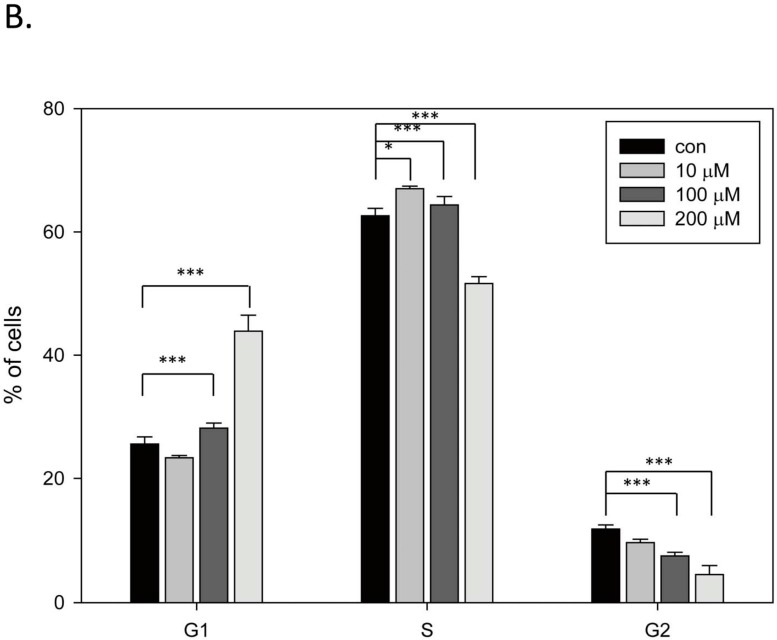
Capsaicin induces cell cycle arrest. TSGH cells were treated with capsaicin or ethanol for 18 h. (**A**) Aliquots of cell lysates were separated by SDS-PAGE and analyzed by western blotting. β-actin was used as an internal control. The intensity of a protein band was quantified by Gel-Pro Analyzer software 3.1. The resultant values of protein expression were normalized to those of actin. Values (mean ± SE) are from three independent experiments (*** *p* <0.001); (**B**) Cells were assayed for cell cycle phase. The graphs are representative of three independent experiments. Values (mean ± SE) are from three independent experiments. The percentage of cells in G1 phase was significantly higher in cells treated with 100 and 200 μM capsaicin compared to cells in the control group by one-way ANOVA with LSD (* *p* < 0.05, *** *p* < 0.001).

To further assess the anti-proliferative effect of capsaicin, we examined changes in the cell cycle distributions. Western blot analyses revealed that capsaicin downregulated tNOX expression, and further revealed downregulation of phosphorylated Rb and cyclin D, which are involved in cell cycle progression (Figure 3A). Cell cycle determination also confirmed that capsaicin significantly enhanced cell arrest at G1 phase at 100 and 200 μM (Figure 3B).

### 2.3. Capsaicin Downregulates tNOX and Reduces Cell Migration

**Figure 4:**
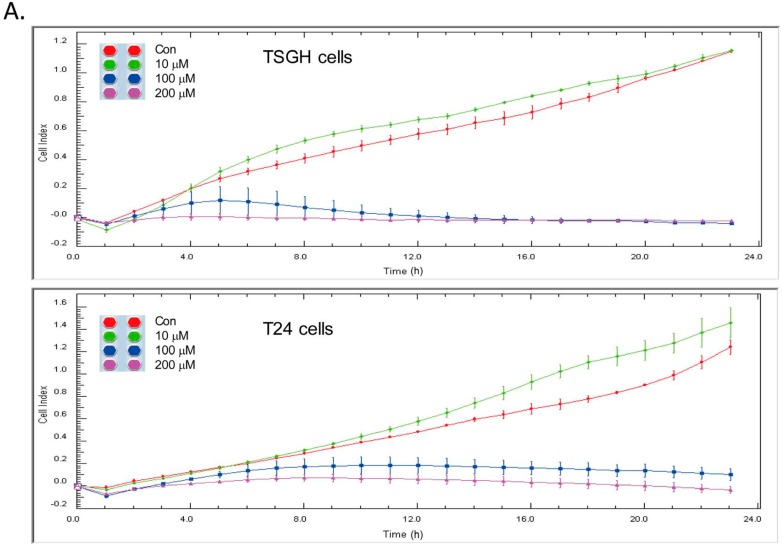

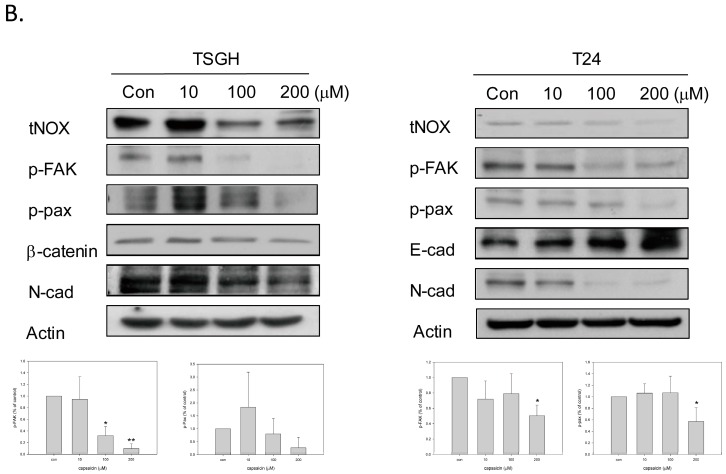
Capsaicin attenuates cell migration. (**A**) Dynamic monitoring of cell migration using impedance technology, as described in Materials and Methods. Shown are normalized cell index measured over 23 h; (**B**) Cells were treated with capsaicin or ethanol for 18 h. Aliquots of cell lysates were separated by SDS-PAGE and analyzed by western blotting. β-actin was used as an internal control. The intensity of a protein band was quantified by Gel-Pro Analyzer software 3.1. The resultant values of protein expression were normalized to those of actin. Values (mean ± SE) are from three independent experiments (* *p* < 0.05, ** *p* < 0.01).

Given that gain- and loss-of-function approaches showed that the expression level of tNOX is relevant to cell migration [27,31], we used cell impedance measurements to examine the effect of capsaicin on the migration of bladder cancer cells. We found that 100 and 200 μM capsaicin effectively attenuated cell migration in both TSGH cells and T24 cell lines (Figure 4A). Interestingly, cell migration was somewhat increased with 10 μM accompanied by up-regulation of tNOX expression, that is consistent with our previous findings in HCT116 cells [32]. Mechanistically, 100 and 200 μM capsaicin inhibited ERK downstream targets, paxillin and FAK, which are all important for cell migration regulation (Figure 4B). The attenuated cell migration was also associated with the downregulation of transcription factors β-catenin, leading to a decrease in mesenchymal marker N-cadherin and an increase in epithelial marker E-cadherin (Figure 4B).

### 2.4. RNA Interference-Mediated tNOX Depletion Reverses Cancer Phenotypes

**Figure 5:**
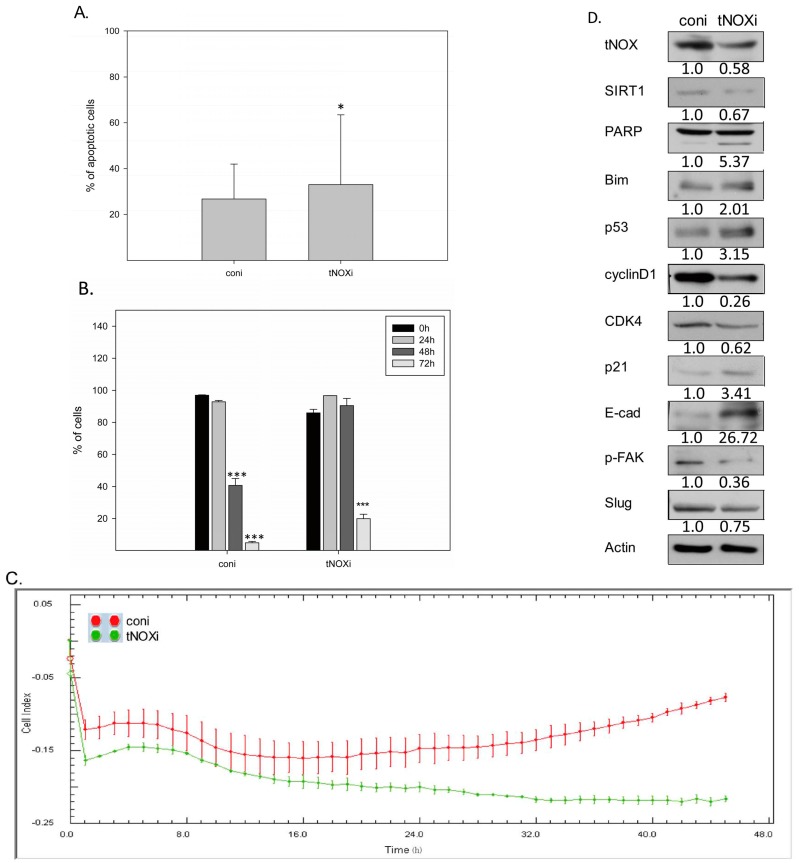
The siRNA-mediated knockdown of tNOX suppresses multiple cancer phenotypes. tNOX was down-regulated by siRNA, and cells were assayed for various cellular functions. (**A**) The percentage of apoptotic cells was determined by flow-cytometry, and the results are expressed as a percentage of apoptotic cells. Values (mean ± SE) are from three independent experiments by *t*-test (* *p* < 0.05); (**B**) Cell division was analyzed by CMFDA staining of tNOX-knockdown and control cells. The presented values (mean ± SE) represent at least three independent experiments by one-way ANOVA with LSD (* *p* < 0.05); (**C**) Dynamic monitoring of cell migration using impedance technology, as described in Materials and Methods. Shown are normalized cell index measured over 45 h; (**D**) Aliquots of cell lysates were separated by SDS-PAGE and analyzed by western blotting; β-actin was used as an internal control. Representative images from three experiments are shown.

Next, we used RNA interference to examine whether tNOX is essential for cancer phenotypes of TSGH cells. Indeed, tNOX depletion was found to significantly increase spontaneous apoptosis (Figure 5A), increase the doubling time (Figure 5B), and decrease cell migration (Figure 5C). We also observed downregulation of SIRT1 in these tNOX-knockdown cells (Figure 5D). The results from protein analyses (Figure 5D) further supported the notion that tNOX knockdown-mediated SIRT1 downregulation reduces the cancer phenotypes of these cultured bladder cancer cells, demonstrating an increase in levels of Bim, caspase 3-direct PARP cleavage, and p53 (enhanced apoptosis), changes in levels of cyclin D, CDK4, and p21 (prolonged cell cycle progression), an increase in E-cadherin while FAK and slug are downregulated (attenuated cell migration).

## 3. Discussion

Among the various signaling pathways involved in capsaicin-mediated cellular responses, oxidative stress has received research attention because it leads to apoptosis [16,17,18,19]. Although mitochondrial ROS generation is thought to be the major source of cellular oxidative stress, other sources may factor into capsaicin-induced apoptosis. For example, the suppression of cyclooxygenase (COX), a ROS-generating enzyme, was shown to be involved in capsaicin-induced apoptosis of human neuroblastoma cells [33]. The plasma membrane-resident NADPH oxidase also responds to capsaicin, and the ROS generated by this enzyme are essential to the capsaicin-induced apoptosis of HepG2 human hepatoblastoma cells [18]. Furthermore, capsaicin exerts its apoptotic activity through overexpression of transient receptor potential vanilloid type 1 (TRPV1), the most-often mentioned cationic channel protein targets of capsaicin, in urothelial carcinoma cells [34,35]. The capsaicin-mediated TRPV1 activation also generates ROS production, mitochondrial dysfunction, and apoptosis in T24 cells [36]. In this study, we present evidence demonstrating that tNOX contributes to capsaicin-mediated apoptosis and is important for capsaicin-induced suppression of cancer phenotypes. Given that tNOX catalyzes the oxidation of NADH to oxidized NAD^+^, the depletion of tNOX reduces NAD^+^ generation and attenuates NAD^+^-dependant SIRT1 deacetylase activity, which is involved in an array of cellular functions, is equally important for capsaicin-induced enrichment in apoptosis and suppression of cell proliferation and migration and apoptosis.

SIRT1, which belongs to the sirtuin protein family, is well conserved in all species and is thought to be involved in cellular functions ranging from epigenetic regulation to metabolism and stress response [37]. The deacetylase activity of SIRT1 on p53 has emerged as a major regulator of apoptosis in stressed cells [38,39]. However, the effect of capsaicin on SIRT1 had not been thoroughly examined in previous studies. We recently reported that capsaicin decreases the intracellular NAD^+^/NADH ratio, thereby reducing SIRT1 activity and p53 acetylation, and ultimately triggering apoptosis in A549 human lung cancer cells [28]. Here, we report that capsaicin-mediated tNOX suppression and experimental knockdown of tNOX reduce SIRT1 expression in a bladder cancer cell line. It has been suggested that SIRT1 is negatively regulated by microRNAs-34a, and that p53 enhances microRNA-34a expression to promote apoptosis [40,41]. Although not yet thoroughly tested in this system, we speculate that the activation of p53 by capsaicin exposure or tNOX depletion might upregulatemicroRNA-34a, and that this might lead to SIRT1 downregulation. Our results also showed that the apoptotic and downstream target of FOXO3 (a renowned downstream target for SIRT1), is enhanced in capsaicin-exposed TSGH cells. Consistent with our results, Wang et al. have reported that FOXO3-induced Bim upregulation is essential for traditional Chinese medicine rhubarb-mediated apoptosis [30].

Our functional and protein assays showed that the capsaicin-induced attenuation of SIRT1 was correlated with reduced cell migration. It was previously reported that the SIRT1 upregulation induced by the carcinogen benzo[a]pyrene (B[a]P), was associated with the upregulation of β-catenin, which drives cell migration, invasion, and even tumorgenesis [42]. Furthermore, the reduced paxillin phosphorylation by ERK inactivation was reported to significantly inhibit cell spreading and migration [43]. Consistent with these, we found that tNOX knockdown possibly affected ERK activation leading to reduced levels of ERK downstream targets, paxillin and FAK, perhaps explaining the observed attenuation of cell migration. Our findings suggest that the reduced SIRT1 expression by tNOX knockdown was found to reduce various cancer phenotypes, and this phenomenon is similar to the way in which sphingosine kinase 1 (Sphk1) and S1P upregulate SIRT1 expression to enhance cell proliferation and migration [44]. Thus, the capsaicin-induced alteration of tNOX and subsequent impact on SIRT1 may exert important effects on multiple cellular pathways. Unfortunately, utilizing capsaicin as an effective cancer strategy has its limitation due to low bioavailability. Thus, it is important to modify, design, or identify new compounds that target tNOX and possess higher bioavailability.

Taken together, our present results show that capsaicin mediates diverse inhibitory effects on the cancer phenotypes of bladder cancer cells, and demonstrate that tNOX depletion exerts similar effects. Furthermore, we provide evidence demonstrating that the capsaicin-induced downregulation of tNOX reduces the expression of SIRT1, which could logically explain the capsaicin-induced deteriorations in multiple cancer phenotypes, including apoptosis, cell cycle progression, and cell migration.

## 4. Materials and Methods

### 4.1. Cell Culture and Reagents

Capsaicin with purity above 95% was purchased from Sigma-Aldrich Corporation (St. Louis, MO, USA). Anti-Bax, anti-Bak, anti-PARP, anti-Bcl-2, anti-p53, anti-phospho-ERK, anti-Bim, anti-phospho-Rb, anti-Rb, anti-Slug, anti-phospho-paxillin, anti-beta-catenin, anti-cyclinD1, anti-p21, anti-acetyl-p53, and anti-SIRT1 antibodies were from Cell Signaling Technology, Inc. (Beverly, MA, USA). Anti-E-cadherin and anti-N-cadherin antibodies were from BD Pharmingen (San Jose, CA, USA). Anti-CDK4 antibody was purchased from Santa Cruz Biotechnology, Inc. (Santa Cruz, CA, USA). Anti-phospho-FAK antibody was from EnoGene Biotech Co, Ltd. (New York, NY, USA). Anti-β-actin antibody was from Millipore Corp. (Temecula, CA, USA). Antisera to tNOX were generated as described previously [27]. Other chemicals were from Sigma-Aldrich Corporation.

TSGH8301 human bladder carcinoma cells were grow in DMEM and T24 human bladder carcinoma cells were grown in RPMI. Media were supplemented with 10% FBS, 100U/mL penicillin and 50 μg/mL streptomycin. Cells were maintained at 37 °C in a 5% CO_2_-95% air-humidified incubator. ON-TARGET plus tNOX (ENOX2) siRNA and negative control siRNA were purchased from Thermo Scientific, Inc. (Grand Island, NY, USA). Briefly, cells were seeded in 10-cm dishes and allowed to attach overnight. The next day, cells were transfected with tNOX siRNA and control siRNA using Lipofectamin RNAiMAX Reagent (Life Technologies, Grand Island, NY, USA) according to the manufacturer’s instructions [45].

### 4.2. Cell Impedance Measurements

Cell impedance technology was used to continuously monitor changes in cell growth. Cells (10^4^ per well) were seeded onto E-plates and incubated for 30 min at room temperature. The E-plates were placed onto the Real-Time Cell Analysis (RTCA) station (xCELLigence System, Roche, Mannhein, Germany), and the cells were grown overnight before exposed to ethanol or different concentrations of capsaicin and cell impedance was measured every hour, as previously described [31].

For continuous monitoring of cell migration, cells (2 × 10^4^ per well) were seeded onto the top chamber of a cell invasion and migration (CIM) plate, which features microelectronic sensors integrated on the underside of the microporous polyethylene terephthalate (PET) membrane of a Boyden-like chamber. After incubation for 30min at room temperature, the CIM plates were placed onto the RTCA station. Cell migration was continuously monitored based on changes in the electrical impedance at the electrode/cell interface.

### 4.3. Apoptosis Determination

Annexin V-FITC Apoptosis Detection Kits were used to determine apoptosis (BD Pharmingen). Briefly, cells treated with ethanol or different concentrations of capsaicin were harvested by centrifugation after trypsinization. Cell pellet was washed with PBS and resuspended in 1× binding buffer. Next, cell pellet was stained with annexin V-FITC (fluorescein isothiocyanate) and also propidium iodide (PI) according to manufacturer’s protocol. The patterns of cell death (necrosis and apoptosis) were analyzed using a Beckman Coulter FC500 flow cytometer and results were expressed as a percentage of total cells.

### 4.4. Measurement of Changes in the Mitochondrial Membrane Potential

Changes in the mitochondrial membrane potential were determined by JC-10 staining according to the manufacturer’s instructions. Briefly, cells were exposed to capsaicin for 3 h, incubated with 10 μM JC-10 for 30 min at 37 °C, and then washed with PBS twice. The changes in the mitochondrial membrane potential were analyzed using a Beckman Coulter FC500 flow cytometer and results were expressed as a percentage of total cells.

### 4.5. Cell Division Assay

RNA interference-targeted tNOX knockdown cells and negative control siRNA-treated cells were labeled by incubating with 5 μM CellTracker Green CMFDA (5-chloromethylfluorescein diacetate; Molecular Probes, Eugene, OR, USA) in fresh medium for 45 min. After treatment, the cells were washed with PBS and trypsinized, and cell division was assessed by flow cytometry as previously described [46].

### 4.6. Measurement of Oxidative Stress

Oxidative stress was determined by measuring the level of hydrogen peroxide generated in the cells with the staining method of 5-(and-6)-carboxy-2′,7′-dichlorodihydrofluorescein diacetate (carboxy-H_2_DCFDA). The basis for this assay is that the nonpolar, nonionic H_2_-DCFDA is cell permeable and is hydrolyzed to nonfluorescent H_2_-DCF by intracellular esterases. In the presence of peroxide, H_2_-DCF is rapidly oxidized to highly fluorescent DCF. At the end of capsaicin treatment, cells (2 × 10^5^) were washed with PBS and incubated with 5 μM H_2_DCFDA in DMSO for 30 min. Cells were collected by trypsinization and centrifugation, washed with PBS, centrifuged at 200× *g* for 5 min and analyzed immediately using a Beckman Coulter FC500 flow cytometer as previously reported [47].

### 4.7. Western Blot Analysis

Cell extracts were prepared in lysis buffer containing 20 mM Tris-HCl pH 7.4, 100 mM NaCl, 5 mM EDTA, 2 mM phenylmethylsulfonyl fluoride (PMSF), 10 ng/mL leupeptin, and 10 μg/mL aprotinin). Volumes of extract containing equal amounts of proteins (40 μg) were applied to SDS-PAGE gels, and resolved proteins were transferred to nitrocellulose membranes (Schleicher & Schuell, Keene, NH, USA). The membranes were blocked with nonfat milk solution for 30 min, then washed, and probed with primary antibody. Membranes were then rinsed with Tris-buffered saline containing 0.1% Tween 20 to remove unbound primary antibody, and incubated with horseradish peroxidase-conjugated secondary antibody for 2 h. The membranes were rinsed again and developed using enhanced chemiluminescence (ECL) reagents (Amersham Biosciences, Piscataway, NJ, USA). The intensity of a protein band was quantified by Gel-pro analysis 3.1 software. The resultant values of protein expression were normalized to those of actin.

### 4.8. Statistics

All data are expressed as the mean ± SD of three or more independent experiments. Comparison between groups was made by one-way analysis of variance (ANOVA) followed by an appropriate *post-hoc* test, such as LSD or *t*-test to analyze the difference. A value of *p* < 0.05 was considered to be statistically significant.
